# Improving Laser Direct Writing Overlay Precision Based on a Deep Learning Method

**DOI:** 10.3390/mi16080871

**Published:** 2025-07-28

**Authors:** Guohan Gao, Jiong Wang, Xin Liu, Junfeng Du, Jiang Bian, Hu Yang

**Affiliations:** 1 Institute of Optics and Electronics, Chinese Academy of Sciences, Chengdu 610209, China; 2 National Key Laboratory of Optical Field Manipulation Science and Technology, Chinese Academy of Sciences, Chengdu 610209, China

**Keywords:** deep learning, laser direct writing, overlay precision, convolutional neural network

## Abstract

This study proposes a deep learning-based method to improve overlay alignment precision in laser direct writing systems. Alignment errors arise from multiple sources in nanoscale processes, including optical aberrations, mechanical drift, and fiducial mark imperfections. A significant portion of the residual alignment error stems from the interpretation of mark coordinates by the vision system and algorithms. Here, we developed a convolutional neural network (CNN) model to predict the coordinates calculation error of 66,000 sets of computer-generated defective crosshair marks (simulating real fiducial mark imperfections). We compared 14 neural network architectures (8 CNN variants and 6 feedforward neural network (FNN) configurations) and found a well-performing, simple CNN structure achieving a mean squared error (MSE) of 0.0011 on the training sets and 0.0016 on the validation sets, demonstrating 90% error reduction compared to the FNN structure. Experimental results on test datasets showed the CNN’s capability to maintain prediction errors below 100 nm in both X/Y coordinates, significantly outperforming traditional FNN approaches. The proposed method’s success stems from the CNN’s inherent advantages in local feature extraction and translation invariance, combined with a simplified network architecture that prevents overfitting while maintaining computational efficiency. This breakthrough establishes a new paradigm for precision enhancement in micro–nano optical device fabrication.

## 1. Introduction

Laser direct writing technology plays a very important role in the fields of integrated circuits, flat panel displays, and micro–nano optical manufacturing [1,2,3,4,5,6]. The precision alignment and overlay process through laser direct writing is an important technique to realize complex and multilayered micro–nano structures in optoelectronic devices [7,8,9,10,11]. The overlay alignment accuracy largely determines the accuracy and performance of the device [12,13,14].

In the mainstream laser direct writing overlay process, high-contrast, sharp-edged patterns (e.g., cross-shaped metal markers) serve as fiducial targets for submicron alignment under microscopy. While miniaturized markers enhance positional resolution, they are more vulnerable to fabrication imperfections. Alignment precision is governed by the following: (i) optical system’s NA/resolution, (ii) mechanical stability/repeatability of air-bearing stages and nanopositioning actuators, and (iii) environmental controls (e.g., dual-frequency laser interferometry and thermal regulation). State-of-the-art systems achieve ≤100 nm absolute positioning accuracy through integrated metrology and motion control architectures. However, stochastic overlay errors (>500 nm peak) arise from defects in fiducial patterns (e.g., edge roughness, partial occlusion), which compromise feature recognition algorithms. For high-precision micro-/nanophotonic devices (e.g., multi-level diffractive optical elements), mitigating these errors is critical to preserving wavefront fidelity and optical performance. Advanced fiducial design strategies and machine learning-based defect compensation techniques are actively pursued to address this challenge [15,16,17,18,19].

The emergence and rise of deep learning technology have provided new ideas for solving the current process optimization problems in the field of laser precision machining [20,21,22]. Deep neural networks mainly consist of four major types: feedforward neural networks (FNNs), convolutional neural networks (CNNs), recurrent neural networks (RNNs), and generative neural networks (GNNs) [23]. Among them, CNNs are constructed imitating the biological visual mechanism, and the implicit convolutional kernels can extract features from images, thus the CNN is most widely used in deep learning research related to image processing.

To address the problem of excessive alignment errors in laser direct writing overlay alignment, we took the technological advantages of deep learning, considering the randomly distributed defects in optical alignment marks, and proposed a method based on deep learning to improve the accuracy of laser direct writing overlay alignment. This approach has reduced the alignment error in mark recognition by an order of magnitude, providing a new idea for enhancing the alignment accuracy of optical alignment marks and laying a technical foundation for overlay alignment applications with higher precision.

## 2. Methods

**Figure 1:**
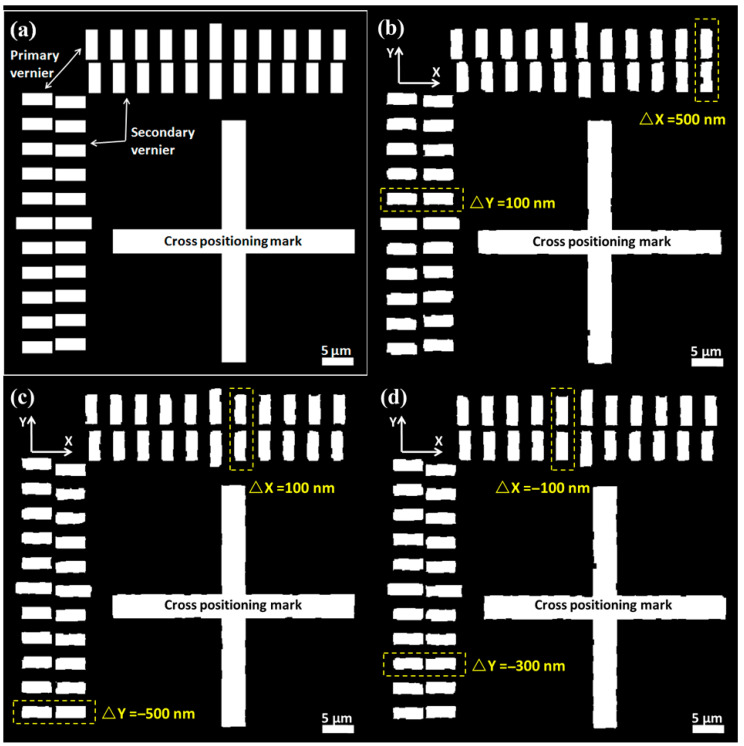
(**a**) Schematic of ideal overlay result with no alignment errors. (**b**–**d**) Schematics of real overlay result with typical alignment errors. The schematics show defective nature of patterns fabricated by photolithography (DWL66+, Heidelberg Instruments, Heidelberg, Germany) with laser wavelength 405 nm and spot size 600 nm. The overlay error stems from multiple sources including optical aberrations, mechanical drift, and fiducial mark imperfections, and we focus on fiducial mark imperfections in this work.

Figure 1 below depicts a typical marking pattern utilized in the laser direct writing overlay process, consisting of a crosshair alignment mark and primary and secondary verniers. The crosshair alignment mark serves the purpose of alignment positioning, while the primary and secondary verniers function to indicate alignment errors. During the actual operation process, the crosshair alignment mark and the primary vernier are first fabricated on the substrate surface along with the first layer pattern. Subsequently, the secondary vernier is fabricated on the substrate surface along with the second layer pattern. The deviation between the primary and secondary verniers can then reflect the overlay alignment error between the two layers. Figure 1a illustrates the ideal overlay process where there is no alignment error between the primary vernier and the secondary vernier. However, Figure 1b–d depict the realistic scenario where randomly distributed alignment errors occur between the primary and secondary verniers during the overlay process. Apart from the resolution errors of the optical system and image processing system and the ranging errors of interference caused by ambient temperature drift, the sources of these alignment errors are mainly caused by the defects in the production of positioning marks, including line width errors, line edge roughness, sand holes, burrs, etc. The main reasons for these defects include defects in coating quality, nonuniform exposure dose of photolithography, and inhomogeneous distribution of development and wet etching rates.

**Figure 2:**
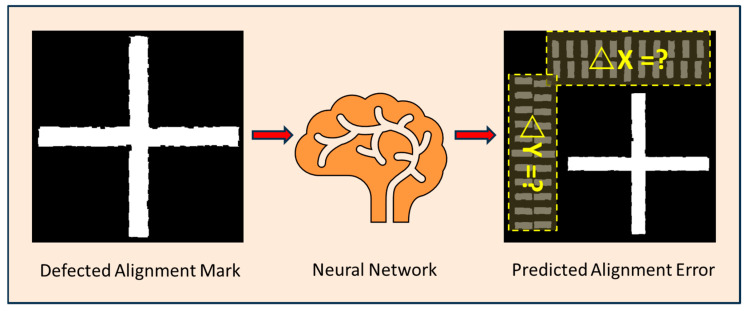
Concept diagram of predicting alignment error of fiducial mark with imperfections via a neural network. The input to the neural network was a grayscale map of crosshair mark with random defects simulating line edge roughness, asymmetric linewidth error, and sand holes. The output was the predicted alignment error. The defects primarily come from process imperfections including exposure nonuniformity, asymmetric development, and wet etching.

Due to the presence of these defects, the image processing system will generate alignment errors when identifying and locating alignment marks. Typical alignment errors are shown in Figure 1b–d, with errors within ±500 nm in both the X and Y directions, and the absolute error depends on the severity of the marker defects. The main innovation of this work lies in the adoption of deep learning methods to restore defective positioning markers to their defect-free state. The principle of this method is illustrated in Figure 2, where the input to the neural network is a grayscale image of a crosshair positioning marker with defects such as linewidth errors, rough edges, and sand holes. The output of the neural network is the alignment error, which represents the difference between the predicted true coordinates of the center position of the crosshair positioning marker and the measured coordinates.

**Figure 3:**
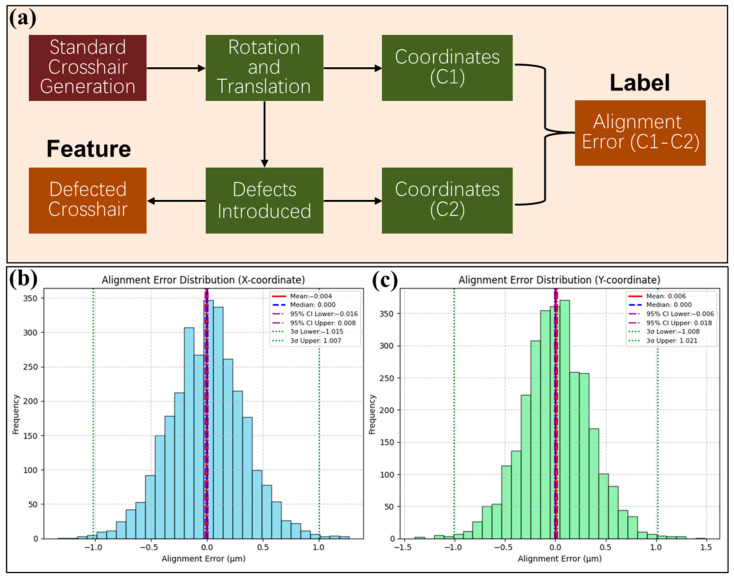
(**a**) Diagram of training data generation method. Firstly, random translations and rotations are applied to a standard crosshair grayscale image, and the center coordinates (c1) of the crosshair at this time are recorded. Subsequently, various defects are introduced by randomly eroding the crosshair, and the center coordinates (c2) at this stage are noted. The difference between c1 and c2 (representing the alignment error) is saved as the dataset label. Finally, the crosshair grayscale images with translations, rotations, and defects are saved as the dataset features. (**b**) Labeled alignment error distribution for the X coordinate in the test set (3000 data). (**c**) Labeled alignment error distribution for the Y coordinate in the test set (3000 data). The 3σ value of alignment error for both coordinates is near 1 μm.

Firstly, FNNs with different structures were built for experiments. In order to obtain sufficient training data, this paper referred to the real crosshair marking defects and used a notebook computer (with Intel i5-12500H 2.50 GHz CPU and 16.0 GB RAM, Santa Clara, CA, USA) to generate 60,000 sets of training data, 3000 sets of validation data, and 3000 sets of test data. Among them, the training data were used to optimize the weight parameters of the neural network through the backpropagation algorithm. The validation data were used for model tuning and to avoid overfitting. The test data was used to evaluate the generalization ability of the model. The data generation method, shown in Figure 3, was based on the standard crosshair gray image, with random translation and rotation generated. The center coordinates of the defect-free crosshair (C1) at this time were recorded. Then, the crosshair was randomly eroded to simulate various defects and coordinates of the defective crosshair (C2) were obtained by pixel calculation. Finally, the crosshair gray images with translation, rotation, and defects were saved as the dataset features, and the difference between C1 and C2 was saved as dataset labels.

**Figure 4:**
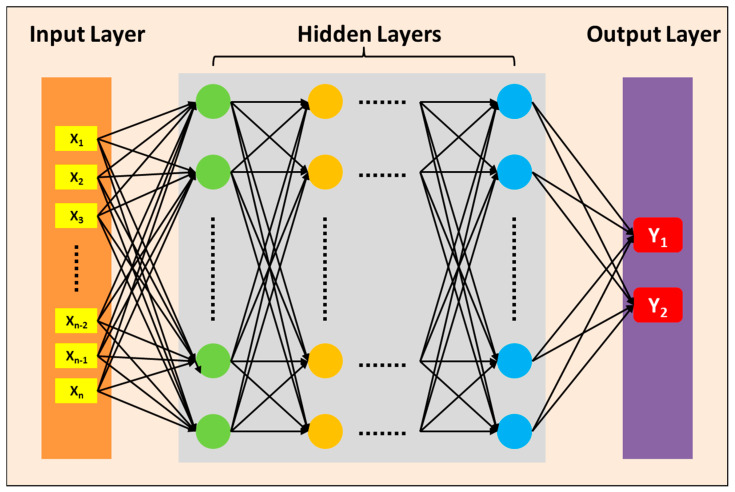
The structure of the FNNs, consisting of an input layer, several hidden layers, and an output layer. The input layer data corresponds to a one-dimensional vector of the crosshair gray image, while the output layer data represents the predicted X and Y coordinates errors of the crosshair center by the neural network.

The structure of FNNs is illustrated in Figure 4, comprising an input layer, several hidden layers, and an output layer. The input layer data consists of one-dimensional vectors corresponding to the grayscale crosshair images, while the output layer data represents the predicted X coordinate error and the Y coordinate error of the crosshair center by the neural network. The process of using an FNN to predict the ideal center coordinates of a defective image relies primarily on its network structure and training data. The defective image is converted into a numerical form suitable for FNN processing and input into the input layer. In the hidden layers, the FNN transforms and extracts features from the input data through fully connected connections, utilizing sufficient hidden layers and neurons to learn and represent the overall features of the image. In the output layer, the FNN performs a regression task, outputting the predicted center coordinates, which are based on the entire network’s understanding and transformation of the input image. Through optimization methods such as the backpropagation algorithm and gradient descent, the FNN continuously adjusts its weights and bias parameters during training to minimize the error between the predicted center coordinates and the actual center coordinates. This requires a large amount of training data to ensure that the FNNs can learn effective feature representations and prediction models. By adjusting the number of neurons in the hidden layers and the number of hidden layers, experiments compare the predictive capabilities of FNNs with different structures.

## 3. Results and Discussions

The FNNs was built using the Keras framework, with ReLU as the activation function, Adam as the optimizer, Mean Square Error (MSE) as the loss function, and a Batch Size of 128. The results were compared after 100 epochs of training. The training results are shown in Table 1 below. It is evident that as the number of hidden layers and neurons increases, the training set loss decreases continuously, from 0.02 to below 0.01. However, the validation set loss remain unchanged above 0.02. Furthermore, with the increase in the number of neurons and hidden layers, overfitting becomes more pronounced. It is assumed that further optimizing the structure of the FNNs is unlikely to significantly lower validation loss.

**Figure 5:**
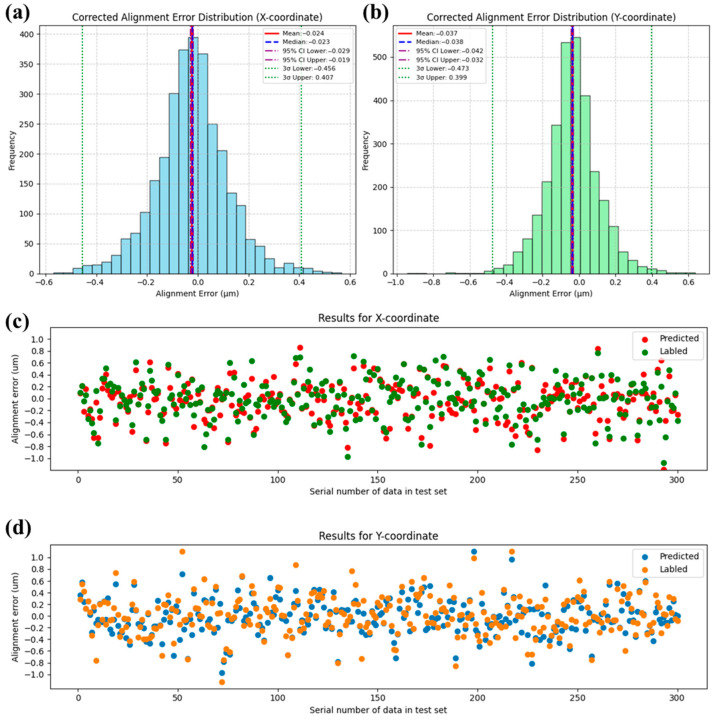
The prediction results of the FNN-6 network (Table 1, No. 6) on the test set are as follows: panel (**a**) represents the corrected alignment error distribution for the X-coordinate, and panel (**b**) represents the corrected alignment error distribution for the Y coordinate. Panel (**c**) shows the first 300 predicted and labeled alignment error for the X coordinate in the test set, and panel (**d**) shows the first 300 predicted and labeled alignment error for the Y coordinate in the test set.

To demonstrate the model’s prediction capability on the test set, we showed statistical distribution of both labeled and predicted alignment error for 3000 results in the test set (Figure 5a,b), and first 300 results of predicted and actual marker coordinates (more data would make the graph less clear) in Figure 5c,d. Statistical results show that the corrected alignment error, obtained by subtracting the predicted alignment error from the labeled alignment error, has a 3σ value reduced to less than 50% of the labeled value. This indicates that the FNN model is capable of reducing the alignment error caused by defective marker recognition by more than 50%. The distribution of the first 300 sets of data also intuitively shows that the FNN model can predict the alignment errors caused by random marker defects to a certain extent. Both in the X coordinate and Y coordinate, the predicted trends are correct, but the approximated values are not ideal, especially for some alignment errors with larger magnitudes. We can observe that there are still many points with deviations greater than 100 nm.

**Figure 6:**
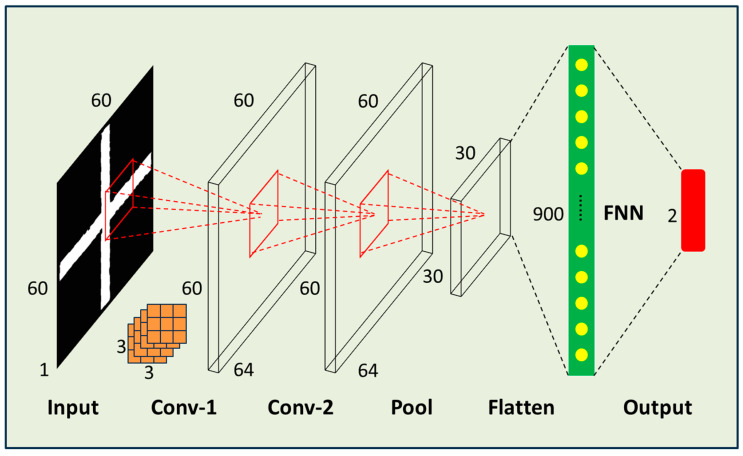
Demonstrated CNN structure with good practical performance. The input is a grayscale image, which undergoes two convolutional processing stages and one pooling stage. After having been flattened, it passes through one fully connected layer before reaching the output layer. The size of the convolutional kernels is 3 × 3.

Based on the FNN, we added convolutional layers and pooling layers to form a Convolutional Neural Network (CNN). The convolutional layers mimic the receptive fields of biological vision to extract features from the input image. Each convolutional kernel extracts a specific feature, and multiple kernels can extract various features. The pooling layers retain the main features while discarding the less important ones, which also compresses the data volume and improves training efficiency. Through multiple convolutional and pooling operations, CNNs can automatically extract key features from the input image and use these features to predict the center coordinates of the graphic. Additionally, CNNs’ translation invariance and end-to-end training approach provide strong support for their performance in graphic center coordinate prediction tasks. By adjusting the network structure and hyperparameters of CNNs, we compared the training loss and validation loss of different models—the result is shown in Table 2 below. A total of eight different network structures’ training results were compared. The first network structure was the most complex architecture, but its training result was the worst in the table. Upon actual prediction, it was found that all coordinates were 0. Therefore, we started to simplify the network structure, reduce the number of convolutional layers, adjust the number of convolution kernels, and remove redundant fully connected layers. Eventually, the eighth network structure performed the best. Figure 6 shows the No. 8 CNN structure that achieved good practical results. The input is a grayscale image, which is processed through two convolutional layers and one pooling layer, then converted into a 1D vector and passed through a fully connected layer to the output layer. The input grayscale image has a size of 60 × 60 × 1; the convolutional kernels have a size of 3 × 3; the pooling layer has a size of 2 × 2; the activation function is ReLU; the optimizer is Adam; the loss function is Mean Squared Error (MSE); and the output layer predicts the positioning marker coordinate error.

The initial design of this network structure was inspired by the classic VGG network structure, which uses more convolutional layers with more neurons and additional fully connected layers. However, practical tests showed that this simple CNN structure outperformed the more complex VGG-like structure. The reason may be that this task involves a limited number of features. The typical VGG network structure is deeper and uses too many fully connected neurons (over 1000), ultimately leading to severe overfitting.

**Figure 7:**
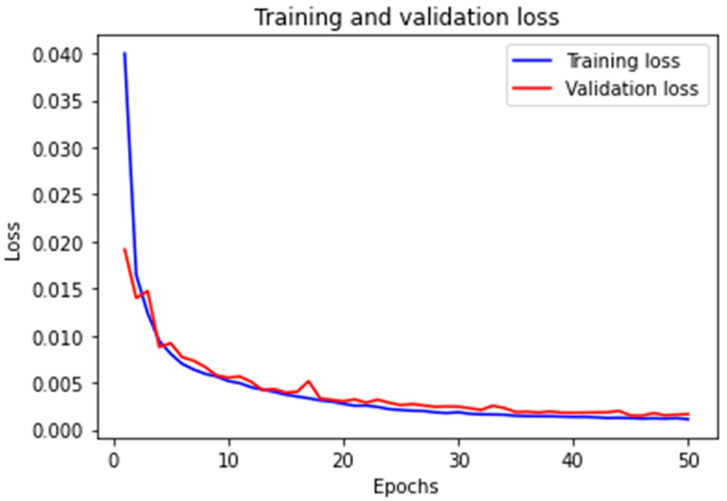
Training and validation loss of the CNN. After 50 epochs, the training loss reaches 0.0011, and the validation loss reaches 0.0016. The loss function is an MSE.

This structure efficiently extracts relevant features from the grayscale image through the convolutional layers, reduces the dimensionality and retains important features through the pooling layer, and finally maps these features to the predicted output through the fully connected layer. The use of 3 × 3 convolutional kernels allows for the extraction of detailed spatial information while maintaining a manageable computational load. The training and validation loss of the CNN model after 50 epochs of training are shown in Figure 7 below. The model achieved convergence with a MSE training loss of 0.0011 and validation loss of 0.0016, which represents a marked improvement over the prior FNN baseline (showing 90% reduction in error). The baseline VGG-style network (five convolutional layers, three fully connected layers) required 15.5 billion FLOPs per inference due to its deep structure with 512-channel convolutions and high-dimensional fully connected layers (4096 nodes). Our streamlined architecture reduces this to 3.8 billion FLOPs through three strategic optimizations: (1) Layer pruning eliminating 60% of convolutional layers (5→2) and 67% of FC layers (3→1); (2) channel compression limiting maximum feature maps to 64 channels (vs 512 in VGG); and (3) implementation of 2 × 2 pooling that progressively halves spatial dimensions from 60 × 60 to 15 × 15. These architectural changes achieved 99.7% parameter reduction (138 M→0.35 M) while maintaining feature extraction capability through preserved 3 × 3 convolutional kernels. Empirical validation on an Intel i5-12500H system confirmed proportional resource savings-training time per epoch decreased 75% (18→4.5 min); GPU memory usage dropped 76.5% (9.8→2.3 GB); and model storage shrank 99.7% (528 MB→1.4 MB). The FLOPs reduction (75.5% theoretical) and actual runtime improvements (75% measured) demonstrate strong correlation, proving the optimization’s effectiveness. This efficiency gain enables real-time defect correction on consumer-grade hardware while achieving sub-100 nm precision, resolving the critical throughput-precision tradeoff in laser direct writing systems.

**Figure 8:**
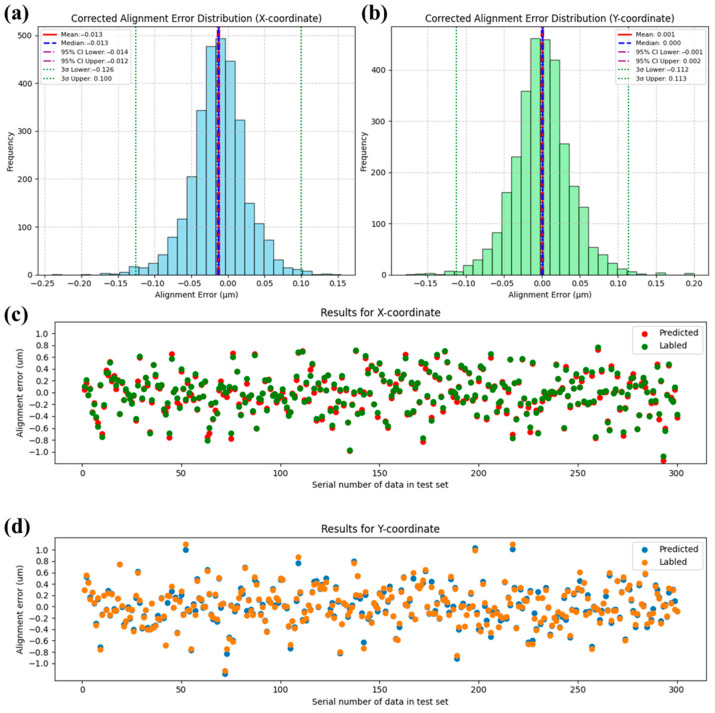
The prediction results of the CNN-8 network (Table 2, No. 8) on the test set are as follows: panel (**a**) represents the corrected alignment error distribution for the X coordinate, and panel (**b**) represents the corrected alignment error distribution for the Y coordinate. Panel (**c**) shows the first 300 predicted and labeled alignment error for the X coordinate in the test set, and panel (**d**) shows the first 300 predicted and labeled alignment error for the Y coordinate in the test set.

The optimized CNN architecture achieved a mean squared error (MSE) of 0.0015 on the test set, demonstrating superior alignment prediction accuracy compared to conventional FNN approaches. As illustrated in Figure 8, statistical results show that the corrected alignment error, obtained by subtracting the predicted alignment error from the labeled alignment error, has a 3σ value reduced to less than 10% of the labeled value. This indicates that the CNN model is capable of reducing the alignment error caused by defective marker recognition by more than 90%. The distribution of the first 300 sets of data also intuitively shows that the CNN model can predict the alignment errors caused by random marker defects to a greater extent than its FNN counterpart. Both in the X coordinate and the Y coordinate, the predicted trends are correct, and the approximated values are very close, even for some alignment errors with larger magnitudes. We can observe that there are few points with deviations greater than 100 nm. This performance stems from the network’s hierarchical feature extraction mechanism: 3 × 3 convolutional kernels systematically capture critical spatial patterns (edge gradients, texture variations) through localized receptive fields, while parameter sharing across translationally invariant operations substantially reduces model complexity. The architecture’s shallow topology (two convolutional layers followed by max-pooling and a single fully connected layer) proves optimal for this defect-correction task, balancing sufficient feature abstraction with minimized overfitting risks—a crucial advantage given the limited morphological complexity of alignment markers. Compared to FNN baselines, the CNN reduces trainable parameters by 98.7% through its weight-sharing paradigm while maintaining spatial robustness to marker positioning variations. This design synergy enables precise coordinate regression (σ < 50 nm) with 75% faster inference speeds than deeper networks, effectively resolving the accuracy–efficiency tradeoff in high-precision overlay alignment systems.

## 4. Conclusions

This research demonstrates the effectiveness of deep learning in addressing laser direct writing overlay alignment challenges. Three key findings emerge: (1) CNN architectures significantly outperform FNN counterparts, reducing alignment errors by 90% through localized feature extraction and parameter sharing mechanisms. (2) Simplified network structures (two convolutional + one pooling + one FC layer) achieve optimal performance for this specific task, outperforming complex VGG-like architectures while reducing computational demands by 75%. (3) The developed defect correction model successfully maintains prediction errors below 100 nm, meeting the precision requirements for multi-layer diffractive optical elements. The method’s success is attributed to three innovative aspects: (a) Simulated defect generation strategy mimicking real process imperfections; (b) dual-coordinate error prediction through end-to-end image regression; (c) architecture optimization balancing feature extraction capability and model complexity. Future work should focus on real-time implementation and extension to 3D alignment scenarios. This approach provides a viable solution for next-generation lithography systems requiring sub-100 nm overlay accuracy, with potential applications in advanced semiconductor manufacturing and nano-photonic device fabrication.

## Figures and Tables

**Table 1:** Training results of FNNs with different structures.

No.	Number of Hidden Layers	Number of Neurons	Training Loss	Validation Loss
1	1	64	0.018	0.022
2	3	64	0.016	0.021
3	6	64	0.016	0.022
4	3	128	0.012	0.021
5	3	256	0.009	0.022
6	3	512	0.006	0.020

**Table 2:** Training results of CNNs with different structures.

No.	Network Structure	Training Loss	Validation Loss
1	Conv32 × 2→Pool→Conv64 × 2→Pool→Conv64 × 3→Pool→Conv128 × 3→Pool→Conv128 × 3→Pool→Flatten→Dense2	0.1131	0.1175
2	Conv16 × 2→Pool→Flatten→Dense64 × 2→Dense2	0.0070	0.0106
3	Conv32 × 2→Pool→Flatten→Dense64 × 2→Dense2	0.0060	0.0092
4	Conv32→Conv64→Pool→Flatten→Dense64 × 2→Dense2	0.0059	0.0103
5	Conv32 × 2→Pool→Conv64 × 2→Pool→Flatten→Dense64 × 2→Dense2	0.0050	0.0099
6	Conv16 × 2→Pool→Flatten→Dense2	0.0029	0.0036
7	Conv32 × 2→Pool→Flatten→Dense2	0.0014	0.0021
8	Conv64 × 2→Pool→Flatten→Dense2	0.0011	0.0016

## Data Availability

Data underlying the results presented in this paper are not publicly available at this time but may be obtained from the authors upon reasonable request.
